# Bifocal diffractive lenses based on the aperiodic Kolakoski sequence

**DOI:** 10.1038/s41598-024-64800-3

**Published:** 2024-06-20

**Authors:** Adrián Garmendía-Martínez, Francisco M. Muñoz-Pérez, Walter D. Furlan, Vicente Ferrando, Juan A. Monsoriu

**Affiliations:** 1 Centro de Tecnologías Físicas, Universitat Politècnica de València, 46022 Valencia, Spain; 2 Departamento de Óptica y Optometría y Ciencias de la Visión, Universitat de València, 46100 Valencia, Spain

**Keywords:** Optical physics, Techniques and instrumentation

## Abstract

In this work, we present a new family of Zone Plates (ZPs) designed using the self-generating Kolakoski sequence. The focusing and imaging properties of these aperiodic diffractive lenses coined Kolakoski Zone Plates (KZPs) are extensively studied. It is shown that under monochromatic plane-wave illumination, a KZP produces two main foci of the same intensity along the axial axis. Moreover, one of the corresponding focal lengths is double the other, property correlated with the involved aperiodic sequence. This distinctive optical characteristic is experimentally confirmed. We have also obtained the first images provided by these bifocal new diffractive lenses.

## Introduction

Diffractive lenses are essential components in image-forming setups at visible wavelengths but also at other spectral ranges in the electromagnetic spectrum. For instance, this kind of lenses offers excellent performance with submillimeter wavelengths (THz frequencies)^1^ and with extreme-ultraviolet and X-rays^2^ for the observation of nanostructures. A Zone Plate (ZP)^3,4^ is the simplest diffractive lens characterized by a series of alternating concentric transparent and opaque annular rings distributed periodically along the square of the radial coordinate, so the area of each annular zone is a constant. Under the paraxial approximation, this zone configuration produces a series of convergent and divergent spherical waves by diffraction when the lens is illuminated by a monochromatic plane wave, hence generating a series of real and virtual foci along the optical axis. To improve diffraction efficiency, ZPs with a binary phase distribution of zones^5,6^ and ZPs with a sawtooth profile (known as kinoform lenses)^7,8^ were proposed. It was theoretically demonstrated that the latter configuration allows concentrating all the energy in a single focus for the design wavelength. Photon Sieves^9–11^ have been proposed to improve the spatial resolution of ZPs. In this application, the transparent annular zones of the amplitude ZPs are replaced by a disjoint set of holes, apodizing in this way the higher order diffraction foci. The combination of Photon Sieves with intracorneal inlays generates a novel alternative for presbyopia treatment^12,13^.

In this framework, we proposed the first aperiodic ZP^14^ of the scientific literature characterized by a distribution of zones following the fractal structure of the Triadic Cantor Set. The resulting fractal diffractive lens produces an axial distribution of self-similar foci when illuminated with a parallel wave front. In subsequent works, our designs were extended to other fractal sets^15,16^ and geometries^17,18^. These fractal ZPs were experimentally characterized as image forming systems, presenting a reduced chromatic aberration and a great depth of field compared to conventional diffractive lenses^19,20^. We have also reported the first experimental results of fractal ZPs focusing capabilities in the terahertz domain^21^. To improve the diffraction efficiency of these fractal lenses, Fractal Photon Sieves^22,23^ were proposed as amplitude elements. On the other hand, the so-called Devil’s Lenses^24,25^ were designed as phase structured fractal lenses being the basis of new fractal intraocular^26,27^ and contact^28,29^ lenses.

Along with the fractals elements, different aperiodic sequences^30,31^ have been employed to design new diffractive lenses with interesting focusing and imaging properties. ZPs based on the Fibonacci^32,33^ and the m-Bonacci^34,35^ sequences are intrinsically bifocals, being the ratio of the two main focal distances related to the generalized golden mean. Thue-Morse ZPs^36,37^ combine the properties of Fibonacci and fractals ZPs producing two main self-similar foci with extended depth of focus along the optical axis. Other aperiodic mathematical generators with which multifocal diffractive lenses have been designed are the he Walsh functions^38^, the precious mean sequence^39^, and the silver mean sequence^40^.

In this paper, we present a new family of aperiodic ZPs arranged according to the rules of the Kolakoski sequence^41^. This self-generating sequence has been applied in several branches of science and engineering, as for example in the context of photonic^42^ and magneto-photonic^43^ crystals, polymer science^44^, nanophotonic waveguides^45^, and applied mathematics^46^, among others. Here we present the first diffractive lenses based on this formalism and an analytical expression for the transmittance function is derived. The focusing properties of Kolakoski ZPs (KZPs) are studied by computing the intensity distribution along the optical axis and the evolution of the diffraction patterns transversal to the propagation direction. We show that a diffractive lens constructed according to the Kolakoski sequence is intrinsically bifocal. The corresponding foci are located at given axial positions correlated with the involved self-generating aperiodic sequence. This property is experimentally verified obtaining a very good agreement with the theoretical prediction computed numerically. The first experimental images produced by this kind of aperiodic structures as bifocal diffractive lenses are also reported.

## Methods

### The Kolakoski sequence

In Mathematics, the so-called “run-length sequence” of a given sequence is itself the sequence formed by those positive integers that indicate the number of elements of equal consecutive symbols in the sequence. For example, the run-length sequence of ABBABBBAABAAABB is 12132132 because the first A appears once, the next B terms appear twice, the next A term appears once, the next B terms appear 3 times, and so on.

The Kolakoski sequence^41^, which we consider here, is an aperiodic sequence, which is identical to its own run-length sequence. In mathematical terms, this sequence can be generated from a seed $K_1=\{1,2\}$. The successive elements of the sequence, $K_S$, are obtained from the previous order, $K_{S-1}$, by applying the substitution rule to the *j*-th element of $K_{S-1}$ in the flowing way: $1\rightarrow 1$ and $2\rightarrow 11$ if *j* is an odd number and $1\rightarrow 2$ and $2\rightarrow 22$ if *j* is an even number. Therefore, $K_2=\{1,2,2\}$, $K_3=\{1,2,2,1,1\}$, $K_4=\{1,2,2,1,1,2,1\}$, $K_5=\{1,2,2,1,1,2,1,2,2,1\}$, $K_6=\{1,2,2,1,1,2,1,2,2,1,2,2,1\}$, etc. Note that the run-length sequence of $K_S$ is $K_{S-1}$. For instance, the sequence $K_4=\{1,2,2,1,1,2,1\}$ presents 1 time 1, 2 times 2, 2 times 1, 1 time 2, and 1 time 1, so its run-length sequence is $K_3=\{1,2,2,1,1\}$.

**Figure 1:**
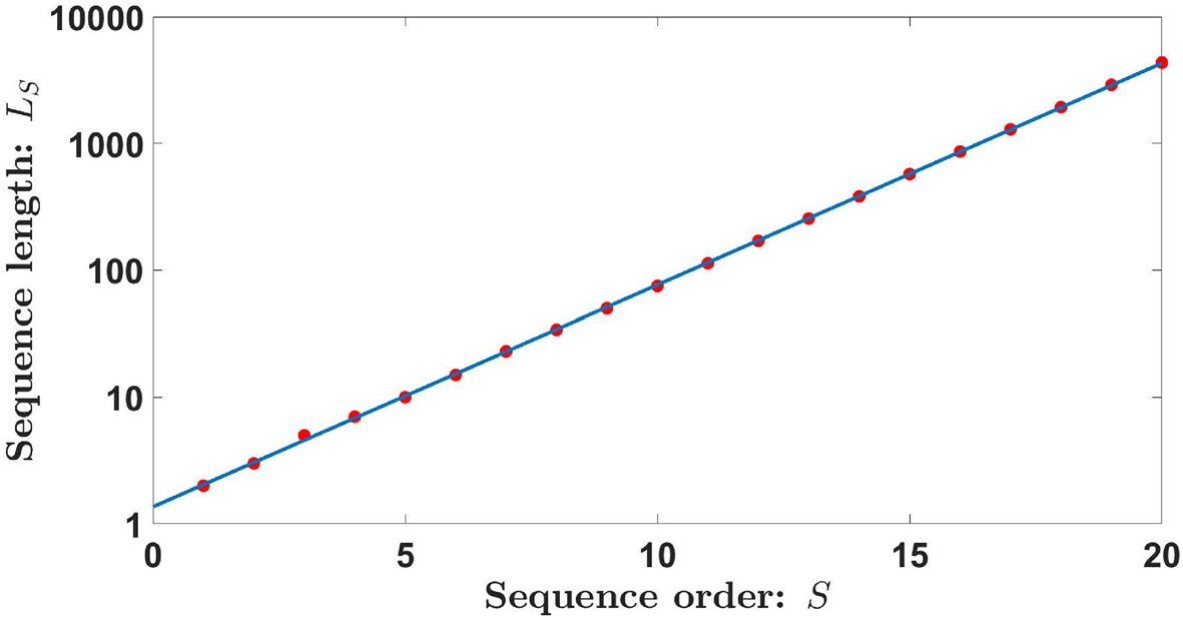
Length $L_S$ of the Kolakoski sequence of order *S* (red points). The result of the linear-logarithmic fit is also included in the figure (blue line).

The red points in Fig. 1 represent the length $L_S$ of the Kolakoski sequence of order *S*, i.e., the total number of elements of the sequence $K_S$. These numbers grow exponentially, $L_S = 2, 3, 5, 7, 10, 15, 23, 34...$, so have been represented on a logarithmic scale.

**Figure 2:**
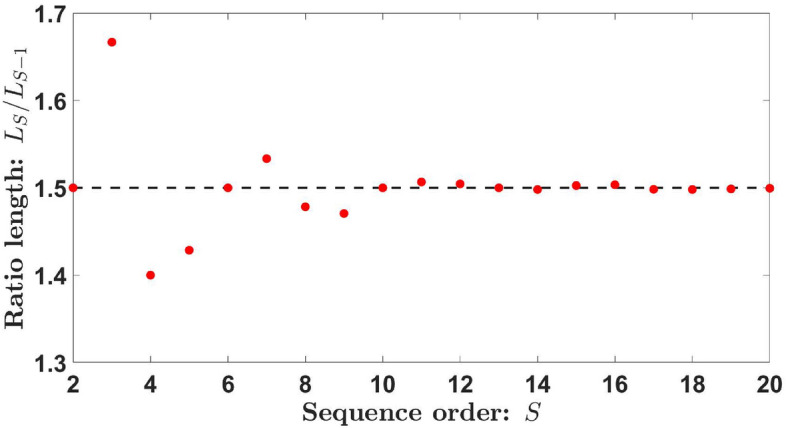
Ratio between the lengths of two consecutive Kolakoski sequences, $L_S/ L_{S-1}$.

By performing a simple linear-logarithmic fitting, a very good approximation for the length of the Kolakoski sequence is $L_S\thickapprox 2\cdot 1.5^{S-1}$ (blue line in Fig. 1). Furthermore, each sequence presents approximately the same number of type “1” and type “2” elements, i.e., $1.5^{S-1}$ elements. On the other hand, if we determine the ratio between the lengths of two consecutive Kolakoski sequences (see Fig. 2), we obtain

1 $$\begin{aligned} \varphi =\lim _{S\rightarrow \infty }\frac{L_S}{L_{S-1}}=3/2, \end{aligned}$$

so the length of the Kolakoski sequence of order *S* is approximately $50\%$ larger than that corresponding to the previous order $S-1$. This value is equivalent to the golden ratio of the Fibonacci sequence^32^, but in this case, we obtain the rational number $\varphi =3/2$. Therefore, the approximated length of the Kolakoski sequence can be expressed as $L_S\thickapprox 2. \varphi ^{S-1}$.

### Kolakoski zone plate design

**Figure 3:**
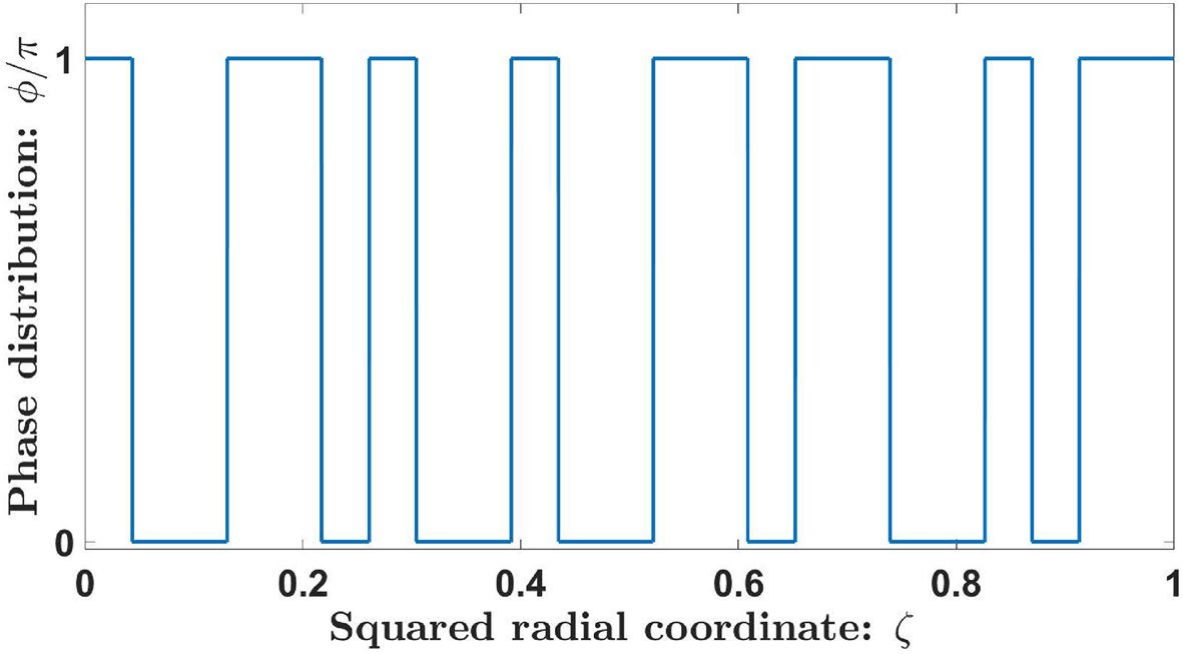
Phase transmission function $\phi _S(\zeta )$ of the KZP of order $S=7$. The associated Kolakoski sequence is $K_7=\{1,2,2,1,1,2,1,2,2,1,2,2,1,1,2,1,1,2,2,1,2,1,1\}$. Note that the phase function takes values $\pi$ or $2\pi$ (phase 0) at the *j*-th sub-intervals of $K_7$ where $K_{7,j}$ is “1” or “2”, respectively.

Based on the Kolakoski sequences, we can design new aperiodic phase binary ZPs. Each one of these sequences, $K_S$, is used to define the phase transmission generating function $\phi _S(\zeta )$, with compact support on the interval $\zeta \in [0,1]$. This interval is partitioned in $L_S$ sub-intervals of length $d_S=1/L_S$. The phase transmittance value, $\phi _{S,j}$, that takes at the *j*-th sub-interval is associated with the element, $K_{S,j}$, being $\phi _{S,j} = \pi K_{S,j}$, so $\phi _{S,j} = \pi$ when $K_{S,j}$ is “1” and $\phi _{S,j} = 2\pi$ or, what is the same, $\phi _{S,j} = 0$ when $K_{S,j}$ is “2” (see Fig. 3).

**Figure 4:**
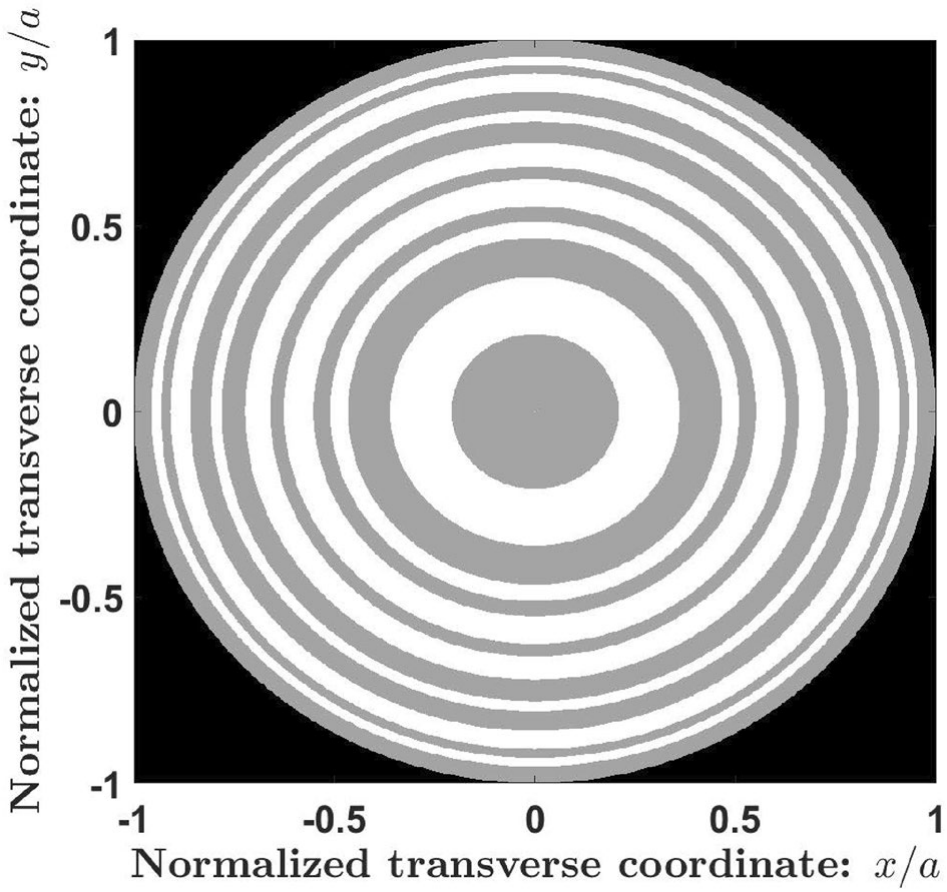
Kolakoski diffractive lens generated from the 1-D function $\phi _7(\zeta )$. Gray and white rings correspond to a phase $\pi$ and 0, respectively.

In mathematical terms, the phase transmission function, $\phi _S(\zeta )$, can be written as:

2 $$\begin{aligned} \phi _S(\zeta )=\sum _{j=1}^{L_S}K_{s,j}.rect\left[ \frac{\zeta -(j-1/2).d_s}{d_s} \right] . \end{aligned}$$

where $\zeta ={(r/a)}^2$ is the normalized squared radial coordinate, *r* is the radial coordinate, *a* is the lens radius, and “*rect*” refers to the rectangular function. Figure 4 shows the corresponding phase distribution of a ZP based on the Kolakoski sequence of order $S=7$. Note that the number of concentric annular zones of a KZP of order *S* coincides with $L_S$. For the case considered in Figs. 3 and 4, the number of zones is $L_7=23$ with approximately the same number of zones with phase $\pi$ (12 zones) and phase $2\pi$ or 0 (11 zones).

### Focusing properties

**Figure 5:**
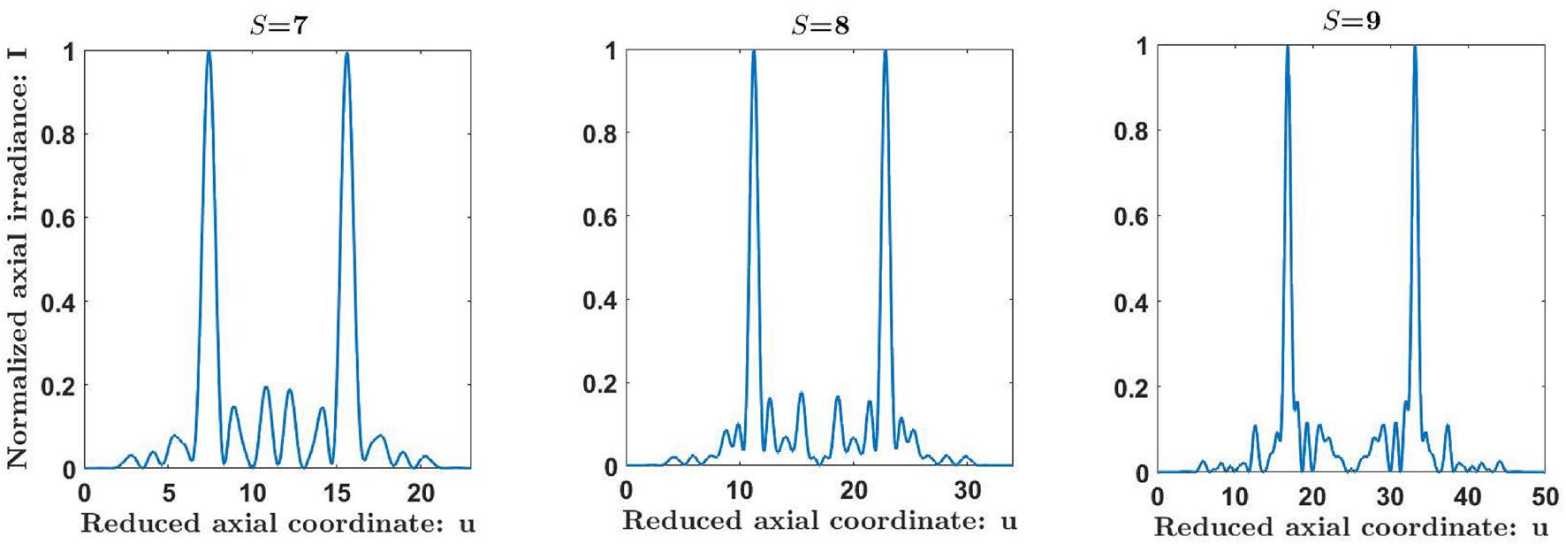
Numerically computed normalized axial irradiances produced by KZPs of orders *S* = 7, 8, and 9.

To evaluate the focusing propierties of the Kolakoski lenses, we have computed the axial irradiance provided by these aperiodic zone plates under a monochromatic plane wave illumination, using the Fresnel-Kirchhoff diffraction theory as^47^:

3 $$\begin{aligned} I_S(u)=4\pi ^2 u^2 \left| \int \limits _0^1 e^{i\phi _S(\zeta )}e^{-i2\pi u\zeta } d\zeta \right| ^2, \end{aligned}$$

where $u=\frac{a^{2}}{2\lambda z}$ is the reduced axial coordinate, *z* is the axial distance from the lens plane to the observation plane, and $\lambda$ is the wavelength of the incident light. If we consider the phase transmittance function given in equation (2), we obtain:

4 $$\begin{aligned} I_S(u)=4\pi ^2 u^2 {d_S}^2 sinc^2[d_Su]\left| \sum _{j=1}^{L_S}e^{i\pi k_{S,j}}e^{-i2\pi ujd_S}\right| ^2, \end{aligned}$$

where $e^{i\pi K_{S,j}}={(-1)}^{K_{S,j}}$ is the transmittance value that takes the Kolakoski lens of order *S* at the *j*-th zone. We have computed the normalized axial irradiance, corresponding to the first diffraction order, provided by KZPs of orders *S* = 7, 8, and 9. The corresponding numbers of phase zones are 23, 34, and 50 for *S* = 7, 8, and 9, respectively. As can be seen in Fig. 5, the axial irradiance distributions, represented against the reduce axial coordinate, *u*, show that the aperiodic ordering of phase zones according to the Kolakoski sequence produces two symmetrical foci around the first diffraction order located at $u_1 = L_S/2\thickapprox \varphi ^{S-1}$. Higher diffraction orders also appear due to the binary nature of the structure (not shown in Fig. 5), so these two foci are periodically replicated along the coordinate *u* with period $u_p=2u_1= L_S\thickapprox 2\varphi ^{S-1}$. Note that the resulting main reduced focal lengths $u_a$ and $u_b$ approximate to $L_S/3\thickapprox 2\varphi ^{S-1}/3$ and $2L_S/3\thickapprox 4\varphi ^{S-1}/3$, so the ratio between the focal distances is $u_b/u_a\thickapprox 2$. Moreover, the ratios $u_1/u_a$ and $u_p/u_b$ approximate to the rational number $\varphi =3/2$ involved in the Kolakoski aperiodic sequence. The higher the order of the sequence, the better these approximations will be. For example, for *S* = 9, the irradiance distribution period is $u_p=50$, the first diffraction order is located at $u_1 = 25$, and the corresponding main focal distances are obtained numerically at $u_a = 16.802$ and $u_b = 33.198$, so $u_b/u_a = 1.976$, $u_1/u_a = 1.488$, and $u_p/u_b = 1.506$.

**Figure 6:**
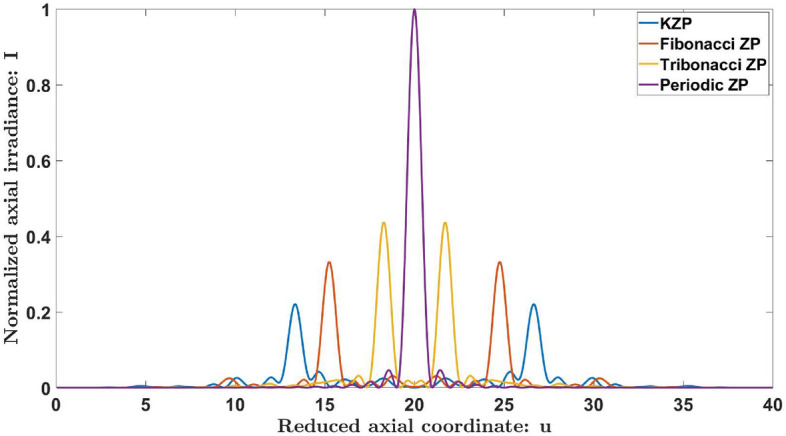
Comparison between the axial irradiances distributions produced by Kolakoski, Fibonacci, Tribonacci and a periodic ZPs.

To contextualize our results within the framework of aperiodic diffractive lenses, the focusing properties of the KZP have been compared with those of the equivalent periodic ZP and other aperiodic intrinsically bifocal ZPs, such as the Fibonacci^32^ ZP and the Tribonacci^34^ ZP. Figure 6 shows the axial irradiance provided by the first 40 zones of these lenses for comparison. These distributions have been normalized to the maximum intensity achieved by the periodic ZP. All these aperiodic lenses split the main focus into a pair of foci with the same axial irradiance, and their separation with respect to the main focal position depends on the properties of the aperiodic sequence. The maximum intensity provided by the KZP is lower compared to the Fibonacci and Tribonacci ZPs, but it also achieves the highest ratio between the focal lengths, with $u_b/u_a\thickapprox 2$ for the KZP, $u_b/u_a\thickapprox 1.615$ for the Fibonacci ZP, and $u_b/u_a\thickapprox 1.189$ for the Tribonacci ZP, providing more options when designing a diffractive lens with specific applications.

### Experimental setup

**Figure 7:**
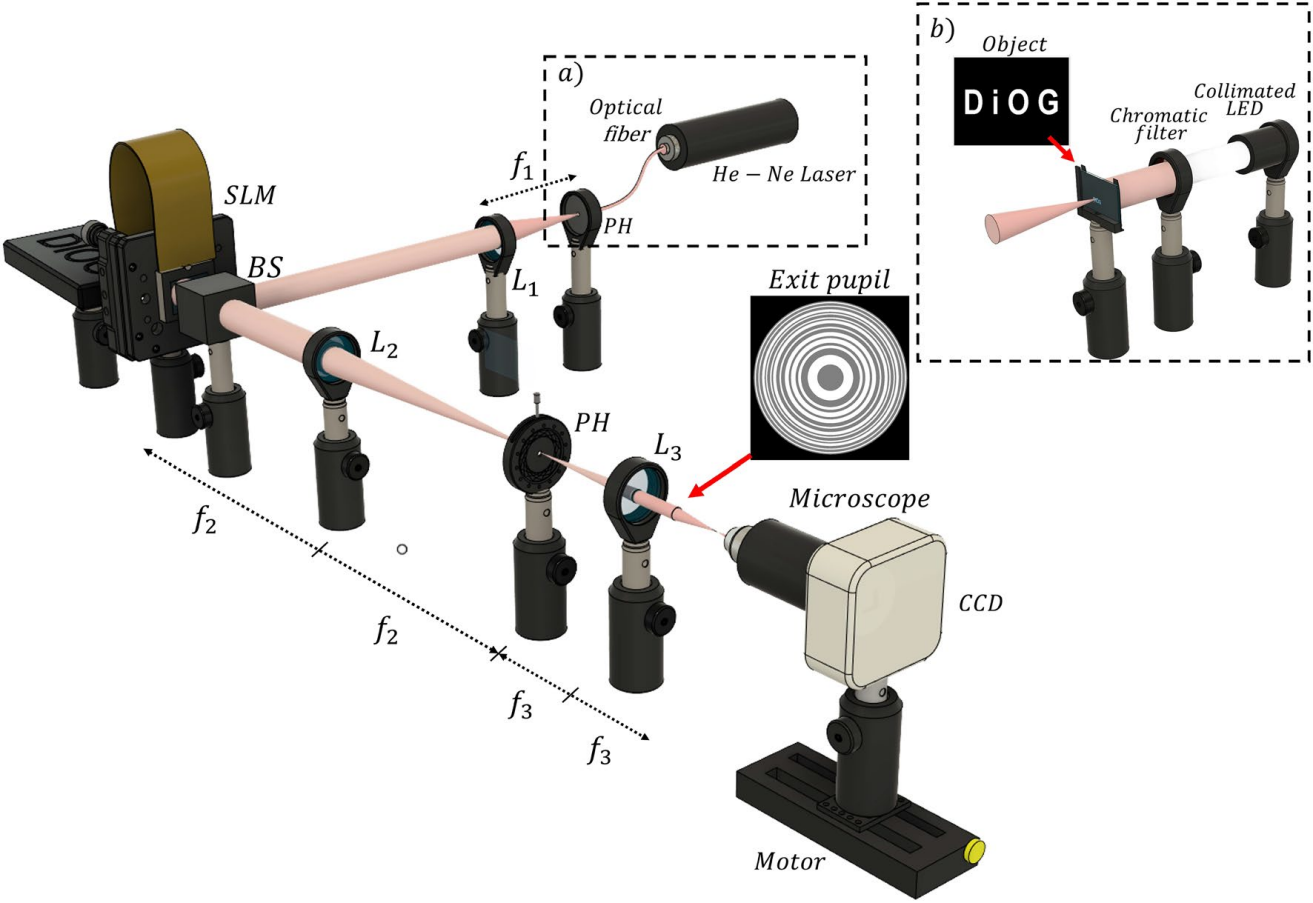
Scheme of the experimental setup used to evaluate (**a**) the focusing and (**b**) imaging properties of the KZP.

The focusing properties of KZPs were tested on the experimental setup shown in Fig. 7. A collimated and linearly polarized beam from an He-Ne Laser ($\lambda = 633$ nm) illuminates the liquid crystal spatial light modulator (SLM) (Holoeye PLUTO, 1920 $\times$ 1080 pixels, pixel size 8 $\mu$m, 8-bit gray-level) where the designed lenses were implemented. The SLM operates in phase-only modulation mode. A linear phase grating was added to the lens modulation; in this way, the addressed signal is deflected to the first diffraction order in the Fourier plane of the lens L3. In addition, the SLM is slightly tilted to correct the linear phase and a pinhole (PH) is positioned at the Fourier plane to eliminate all diffraction orders of the linear phase grating except order +1. The PH also prevents noise from the specular reflection (zero diffractive order) and the pixelated structure of the SLM (higher diffraction orders). Then, the SLM plane is imaged through a telescopic system (L2 and L3). In this way, the studied lens transmittance is projected at the exit pupil plane and its focusing binary profile can be captured along the axis by a camera sensor mounted on a motorized stage.

In order to evaluate the imaging properties of this lens, we modified the previous experimental setup, as illustrated in Fig. 7.b. As illumination source, we replaced the He-Ne laser beam by a collimated LED with a chromatic filter, corresponding to $\lambda = 633$ nm, and a binary object with the letters DiOG (Diffractive Optics Group) (see the inset in Fig. 7).

## Results

**Figure 8:**
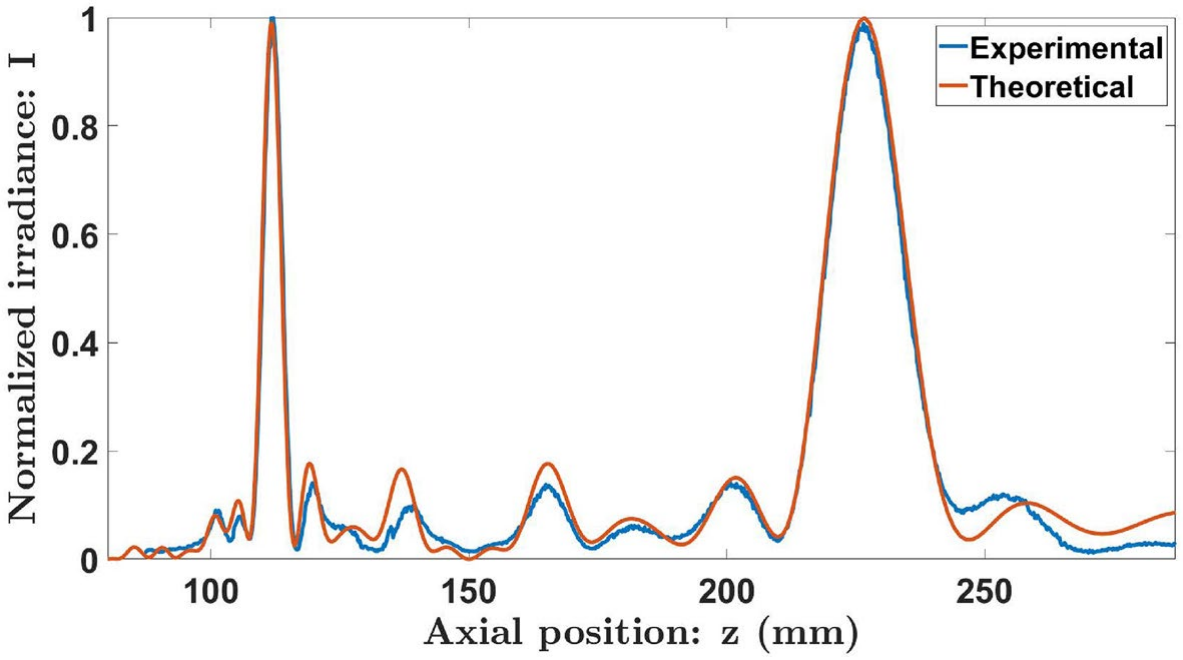
Theoretical and experimental axial irradiance profiles of the Kolakoski lens of order $S=8$. Both of these distributions are normalized with respect to the maximum intensity..

**Figure 9:**
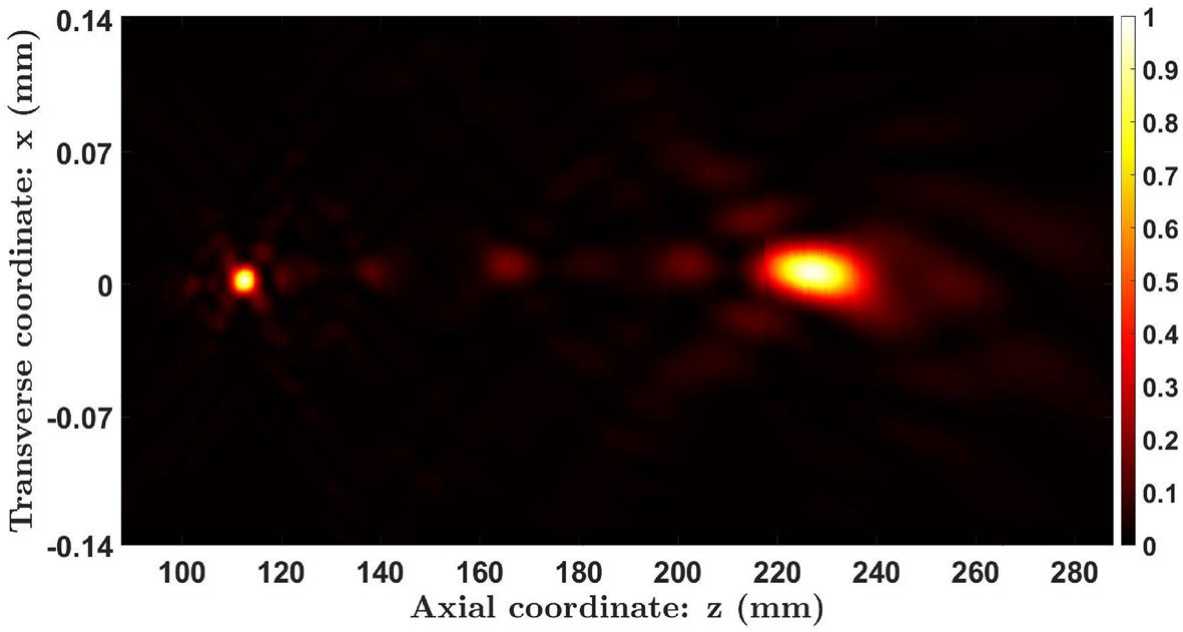
Evolution of the transverse intensity distribution produced by a KZP of order 8.

We assessed the focusing properties of a KZP of order *S* = 8 and radius *a* = 1.80 mm. Figure 8 shows the experimental axial irradiance distribution along with the one obtained numerically using Eq. (3). Both results are in good agreement. It can be seen that the Kolakoski lens provides two foci with very similar intensities whose axial positions are $z_a=226.5$ mm and $z_b=111.7$ mm. The corresponding experimental reduced axial coordinates, $u=\frac{a^2}{2\lambda z}$, are $u_a=11.25$ and $u_b=22.83$, respectively. As predicted from the theoretical analysis, the ratio between the positions of these foci approximates to $\frac{u_b}{u_a}=2.03\thickapprox 2$. Moreover, if we compute the ratios $\frac{u_1}{u_a}$ and $\frac{u_p}{u_b}$, where $u_1=17$ and $u_p=34$ for the Kolakoski sequence of order 8, we can see that they both approximate to $\varphi$: $\frac{u_1}{u_a}\thickapprox 1.5$, $\frac{u_p}{u_b}\thickapprox 1.5$. To provide a more extensive study of the focusing characteristics of the KZP, the transversal irradiance distribution in the *xz* plane was also captured experimentally (Fig. 9). This result confirms the bifocal behavior of the lens as well as the corresponding ratio between its focal lengths.

**Figure 10:**
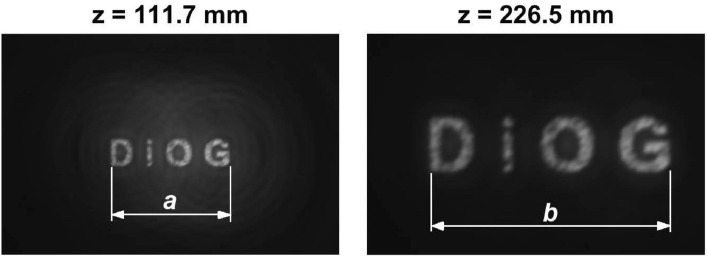
Images provided by a KZP of order 8 at its corresponding focal lengths.

Finally, the monochromatic images for the aforementioned wavelength provided by the KZP were captured at several axial positions in the range [88 mm - 288 mm] (see Supplementary video). As expected, this ZP produces two focused images of the object at positions 111.7 mm and 226.5 mm where the two foci are located (see Fig. 10). Some halos surrounding the DiOG letters in the first focus can be noticed, since the out-of focus images corresponding to the higher diffraction orders, are superimposed to the in-focus image. On the other hand, it can be observed that the second image is double the transverse size of the first one, a property related to the generating aperiodic sequence. In fact, in Fig. 10, the resulting relative sizes of the segments are $a=0.452$ mm and $b=0.914$ mm, satisfying $\frac{b}{a}=2.02\thickapprox 2$.

## Discussion

A diffractive lens based on the aperiodic Kolakoski sequence has been presented and studied both numerically and experimentally. It was shown that a KZP produces two foci along the optical axis being the corresponding focal lengths correlated with the involved aperiodic Kolakoski sequence. The image-forming capabilities of these bifocal lenses were also tested. We believe that the proposed aperiodic diffractive lens could be of benefit across a broad range of applications where conventional ZPs are currently applied, such as X-ray microscopy, THz imaging, and ophthalmology. Our next step is to design kinoform-type diffractive structures based on this sequence. This step would aim to improve the diffraction efficiency of the lens, thus extending its suitability to an even broader spectrum of optical applications.

### Supplementary Information

Supplementary Information.

## Data Availability

All data generated or analysed during this study are included in this article.
